# Additional malignancies shorten overall survival in chronic lymphocytic leukemia irrespective of chromosomal aberrations: A retrospective cohort study

**DOI:** 10.1097/MD.0000000000032906

**Published:** 2023-02-10

**Authors:** Esra Turan Erkek, Eda Aslan

**Affiliations:** a Department of Hematology, Medical Science University Kartal Dr Lutfi Kirdar City Hospital, Istanbul, Turkey; b Department of Internal Medicine, Sindirgi State Hospital, Balikesir, Turkey.

**Keywords:** chronic lymphocytic leukemia, mortality, other malignancies

## Abstract

The aim of this study was to determine the incidence of other malignancies (OMs) in patients with chronic lymphocytic leukemia (CLL) and to identify parameters associated with the occurrence of OMs in addition to CLL. This retrospective cohort study was conducted by examining the records of CLL patients who applied to a tertiary hospital between January 2013 and December 2021. The cases were divided into 2 groups, CLL (n = 107) and CLL + OM (n = 25), according to the presence of additional malignancy. Lymphocyte count (*P* = .014), white blood cell count (*P* = .006), and hemoglobin (*P* = .034) were significantly higher in the CLL group. Rai stage IV percentage (*P* = .015), Binet stage B percentage (*P* = .043), progression, and sepsis percentages (*P* = .008) were significantly higher in the CLL + OM group. Overall survival time was significantly lower in the CLL + OM group (*P* = .032). Most OMs had been diagnosed before CLL (63.64%) in the no-treatment group, while the majority of OMs were diagnosed after CLL (78.57%) in the treatment group (*P* = .032). CLL patients with OM had a more advanced CLL stage, and survival was significantly shorter in these patients. In addition, CLL-associated OM appears to occur more frequently in the post-treatment period.

## 1. Introduction

Chronic lymphocytic leukemia (CLL), characterized by the accumulation of mature, small, monoclonal malignant B lymphocytes in the blood, bone marrow, and lymph nodes, is rather common in the elderly population.^[1]^ With the effective use of modern treatment options, survival has increased in patients with CLL. Due to prolonged survival, CLL patients have evolved into an older patient population with various comorbidities and even secondary malignancies.^[2]^ It has been reported that the rates of other malignancies (OMs) involving the skin, lung, kidney, and prostate increase in patients with CLL.^[3]^ Among the treatment options currently used in symptomatic or high-risk patient groups, in addition to conventional options such as alkylating agents and purine analogs, agents such as monoclonal antibodies, Bruton tyrosine kinase inhibitors, and B-cell lymphoma 2 inhibitors are being utilized.^[4]^ Interestingly, studies have emphasized that various malignancies may occur due to the treatments applied for CLL.^[5–8]^

In the last decade, scores using age, sex, Eastern Cooperative Oncology Group performance, and B2 microglobulin level have been used to evaluate the prognosis of CLL.^[9,10]^ However, these scoring systems are thought to be insufficient in CLL case groups with comorbid diseases and OMs.^[11,12]^ Therefore, new studies are needed to evaluate the clinical features and prognosis of patients with other diseases in addition to CLL.

In this study, we aimed to determine the incidence of OM in patients with CLL, to identify parameters that may be associated with the occurrence of OM in addition to CLL, and to compare the clinical results of these 2 case groups.

## 2. Methods

### 2.1. Study population

This retrospective cohort study was conducted by examining the records of CLL patients who applied to Kartal Dr Lütfi Kirdar City Hospital Hematology Outpatient Clinic between January 2013 and December 2021. Ethical approval for study conduct was obtained from the Clinical Research Ethics Committee of Kartal Dr Lutfi Kirdar City Hospital (Date: 14.10.2020, no: 2020/514/187/8). Informed consent was obtained from all individual participants included in the study.

All patients who were diagnosed with CLL between the relevant dates and were followed up in our hospital were included in the study. Age at diagnosis, sex, chromosomal anomalies, comorbidities, laboratory parameters, presence of splenomegaly, disease stages (Rai stage and Binet stage, both described in Table 1),^[13]^ CLL treatment status, survival time, cause of mortality, and total follow-up time were recorded. The cases were divided into 2 groups, those with CLL and those with CLL + OM, according to the presence of additional malignancy. All analyzed parameters were compared between these 2 groups, while further evaluations were also performed to ascertain the associations between the utilization of CLL treatment and factors such as OM type, OM therapy (chemotherapy or hormonotherapy; radiotherapy), and the timing of OM diagnosis (before, concurrent with, or after CLL diagnosis).

**Table 1:** The Rai and Binet staging systems used in patients with chronic lymphocytic leukemia (21).

Risk stratification	Rai stage	Binet stage
Low risk	0: Lymphocytosis only	A: <3 Lymphadenopathies
Intermediate risk	I: Lymphadenopathy	B: >3 Lymphadenopathies
	II: Organomegaly (splenomegaly/hepatomegaly)	
High risk	III: Anemia (hemoglobin < 11 g/dL)	C: Hemoglobin < 10 g/dL and/or platelets < 100 × 10^9^/L
	IV: Thrombocytopenia (platelets < 100 × 109/L)	

### 2.2. Statistical analysis

All analyses were performed on SPSS v25 (SPSS Inc., Chicago, IL) and were subject to a 2-tailed *P* ≤ .05 significance threshold. Continuous variables’ normality of distribution in respective groups was evaluated with plots (Q-Q and histogram). Descriptive data for continuous variables are depicted with mean ± standard deviation or median (1st quartile–3rd quartile) with regards to the normality of distribution. Relative and absolute frequencies were described for categorical variables. Continuous variables meeting parametric assumptions were compared with the independent samples *t* test, while those not meeting these assumptions were compared with the Mann–Whitney *U* test. Categorical variables were analyzed with the chi-square tests or Fisher exact tests. Overall survival times were calculated with the Kaplan–Meier method and were analyzed with the log-rank test.

## 3. Results

We included 132 patients (48 females and 84 males) in our study; the mean age at diagnosis was 63.92 ± 10.26 (range 39–88) years. A total of 107 (81.06%) patients had only CLL, while 25 (18.94%) had CLL + OM. The most common OMs were breast (20.00%) and colorectal (20.00%) cancers. Twelve (48.00%) patients received chemotherapy/ hormonotherapy for OM and 11 (44.00%) patients received radiotherapy for OM. Ten (40.00%) OMs had been diagnosed before CLL, 1 (4.00%) OM was diagnosed concurrently with CLL, and 14 (56.00%) OMs were diagnosed after CLL. We found no significant differences between the CLL and CLL + OM groups in terms of age at diagnosis, sex, del 17p, del 11q, del 13q, trisomy 12, and comorbidities. White blood cell (WBC) count (*P* = .006), lymphocyte count (*P* = .014) and hemoglobin (*P* = .034) levels were significantly higher in the CLL group than in the CLL + OM group. We found no significant differences the between groups in terms of LDH, beta-2 microglobulin, platelet count, aspartate transaminase, alanine transaminase, and C-reactive protein values (Table 2).

**Table 2:** Summary of patient characteristics with regard to groups.

		Groups		
	Total (n = 132)	CLL (n = 107)	CLL + OM (n = 25)	*P*
Age at diagnosis	63.92 ± 10.26	63.47 ± 10.33	65.88 ± 9.92	.291
Sex				
Female	48 (36.36%)	39 (36.45%)	9 (36.00%)	1.000
Male	84 (63.64%)	68 (63.55%)	16 (64.00%)	
del 17p	12 (9.09%)	9 (8.41%)	3 (12.00%)	.698
del 11q	20 (15.15%)	17 (15.89%)	3 (12.00%)	.764
del 13q	32 (24.24%)	27 (25.23%)	5 (20.00%)	.771
Trisomy 12	15 (11.36%)	13 (12.15%)	2 (8.00%)	.735
Comorbidities	111 (84.09%)	90 (84.11%)	21 (84.00%)	1.000
Diabetes mellitus	24 (18.18%)	17 (15.89%)	7 (28.00%)	.161
Hypertension	44 (33.33%)	36 (33.64%)	8 (32.00%)	1.000
Coronary artery disease	16 (12.12%)	12 (11.21%)	4 (16.00%)	.504
Heart diseases	8 (6.06%)	6 (5.61%)	2 (8.00%)	.646
Renal diseases	8 (6.06%)	8 (7.48%)	0 (0.00%)	.352
COPD	7 (5.30%)	4 (3.74%)	3 (12.00%)	.125
Cerebrovascular disease	4 (3.03%)	3 (2.80%)	1 (4.00%)	.573
Hepatitis	8 (6.06%)	6 (5.61%)	2 (8.00%)	.646
Other	45 (34.09%)	40 (37.38%)	5 (20.00%)	.157
LDH, U/L	218 (185–278.5)	225 (186–286)	215 (163–250)	.233
β-2 Microglobulin, ng/mL	3000 (2158–4052)	2933 (2225–3819)	3640 (2158–4248)	.488
WBC, ×1000/uL	24.06 (13.2–55.5)	26.7 (14.9–63)	16.2 (10.1–30.01)	**.006**
Lymphocyte, ×1000/uL	16 (8.4–42.25)	19 (9.8–50)	11.06 (5.8–25.8)	**.014**
Hemoglobin, g/dL	12.81 ± 2.00	12.99 ± 1.91	12.04 ± 2.23	**.034**
Platelet, ×1000	182.46 ± 76.64	186.42 ± 71.05	165.52 ± 96.95	.318
AST, U/L	21 (17–26)	21 (17–26)	19 (15–26)	.238
ALT, U/L	17.5 (14–25)	17 (14–25)	18 (14–24)	.848
CRP	3.3 (3.03–4.6)	3.3 (3.02–4.3)	3.3 (3.1–5.92)	.337
Splenomegaly	41 (31.06%)	33 (30.84%)	8 (32.00%)	1.000
Rai stage				
0	22 (16.67%)	20 (18.69%)	2 (8.00%)	**.015**
I	55 (41.67%)	42 (39.25%)	13 (52.00%)	
II	33 (25.00%)	30 (28.04%)	3 (12.00%)	
III	10 (7.58%)	9 (8.41%)	1 (4.00%)	
IV	12 (9.09%)	6 (5.61%)	6 (24.00%)	
Binet stage				
A	67 (50.76%)	52 (48.60%)	15 (60.00%)	**.043**
B	42 (31.82%)	39 (36.45%)	3 (12.00%)	
C	23 (17.42%)	16 (14.95%)	7 (28.00%)	
CLL treatment	94 (71.21%)	80 (74.77%)	14 (56.00%)	.105
Mortality	45 (34.09%)	34 (31.78%)	11 (44.00%)	.354
Progression	7 (15.56%)	3 (8.82%)	4 (36.36%)	**.008**
Sepsis/Febrile neutropenia	15 (33.33%)	8 (23.53%)	7 (63.64%)	
Pneumonia	3 (6.67%)	3 (8.82%)	0 (0.00%)	
COVID-19	3 (6.67%)	3 (8.82%)	0 (0.00%)	
Other	10 (22.22%)	10 (29.41%)	0 (0.00%)	
Unknown	7 (15.56%)	7 (20.59%)	0 (0.00%)	
Follow up time, months	59 (33–81)	62 (34–81)	48 (32–78)	.593
Overall survival time, months*	135.98 ± 10.79	147.40 ± 12.65	106.58 ± 15.82	**.032**

Data are given as mean ± standard deviation or median (1st quartile–3rd quartile) for continuous variables according to normality of distribution and as frequency (percentage) for categorical variables.

ALT = alanine aminotransferase, AST = aspartate aminotransferase, CLL = chronic lymphocytic leukemia, COPD = chronic obstructive pulmonary disease, COVID-19 = coronavirus disease 2019, CRP = C-reactive protein, LDH = lactate dehydrogenase, OM = other malignancies, WBC = white blood cell.

* Calculated with Kaplan–Meier method and were given as mean ± standard error.

**Figure 1.**
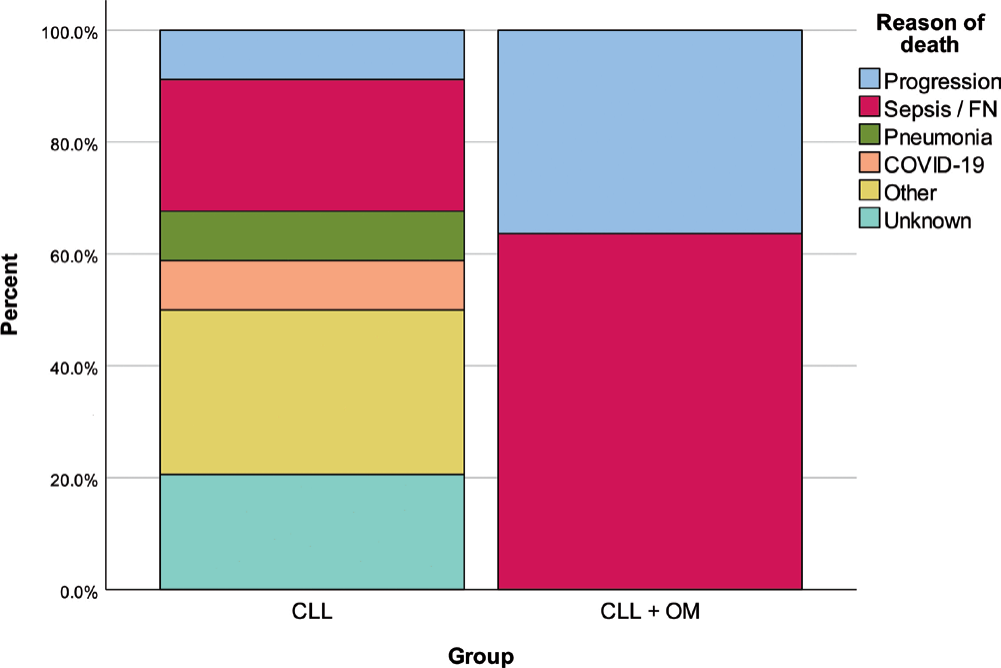
Reasons of death with regard to groups.

Frequency of Rai stage IV was significantly higher in the CLL + OM group than in the CLL group (*P* = .015). Binet stage B percentage was significantly higher in the CLL group than in the CLL + OM group (*P* = .043). There were no significant differences between the groups in terms of splenomegaly and CLL treatment. Thirty-four (31.78%) cases died in the CLL group, while 11 (44.00%) cases had died in the CLL + OM group (*P* = .354). When we evaluated causes of mortality, we found progression (OM progression in all cases) and sepsis percentages were significantly higher in the CLL + OM group than in the CLL group (*P* = .008) (Fig. 1). In addition, overall survival time was significantly shorter in the CLL + OM group than in the CLL group (*P* = .032) (Table 2).

**Figure 2.**
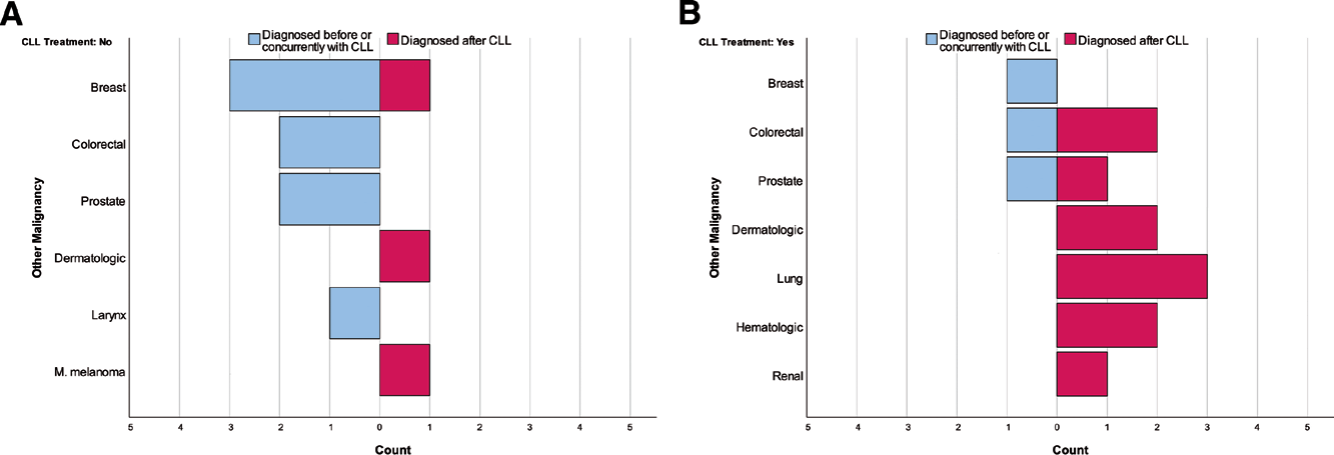
(A) Other malignancies with regard to diagnosis time. (A) CLL no-treatment group. (B) CLL treatment group. CLL = chronic lymphocytic leukemia.

When OM cases were compared with respect to the utilization of CLL treatment, we found that the most common OM was breast cancer (36.36%) in the no-treatment group while colorectal (21.43%) and lung (21.43%) cancers were common in the treatment group. Most of the OMs were diagnosed before CLL (63.64%) in the no-treatment group, while most OMs in the treatment group were diagnosed after CLL (78.57%) (*P* = .032). Assessment of OMs with regard to diagnosis time is shown in Figure 2A and B. We found no significant differences between treatment groups in terms of OM type, chemotherapy/hormonotherapy, and radiotherapy (Table 3).

**Table 3:** Summary of other malignancies with regard to CLL treatment.

		CLL treatment		
	Total (n = 25)	No (n = 11)	Yes (n = 14)	*P*
Other malignancy				
Breast	5 (20.00%)	4 (36.36%)	1 (7.14%)	.257
Colorectal	5 (20.00%)	2 (18.18%)	3 (21.43%)	
Prostate	4 (16.00%)	2 (18.18%)	2 (14.29%)	
Dermatologic	3 (12.00%)	1 (9.09%)	2 (14.29%)	
Lung	3 (12.00%)	0 (0.00%)	3 (21.43%)	
Hematologic	2 (8.00%)	0 (0.00%)	2 (14.29%)	
Larynx	1 (4.00%)	1 (9.09%)	0 (0.00%)	
Malign melanoma	1 (4.00%)	1 (9.09%)	0 (0.00%)	
Renal	1 (4.00%)	0 (0.00%)	1 (7.14%)	
Chemotherapy/Hormonotherapy	12 (48.00%)	6 (54.55%)	6 (42.86%)	.859
Radiotherapy	11 (44.00%)	4 (36.36%)	7 (50.00%)	.689
OM diagnosis time				
Before CLL	10 (40.00%)	7 (63.64%)	3 (21.43%)	**.032**
Concurrent with CLL	1 (4.00%)	1 (9.09%)	0 (0.00%)	
After CLL	14 (56.00%)	3 (27.27%)	11 (78.57%)	

Data are given as frequency (percentage).

CLL = chronic lymphocytic leukemia, OM = other malignancies.

## 4. Discussion

The addition of comorbidities in patients with CLL, which is usually seen in advanced age, may affect clinical outcomes. For this reason, it is valuable to examine the frequency of such comorbidities and to assess possible parameters that affect outcomes.^[14]^ In this study, it was determined that disease stage, cause of mortality, and survival time demonstrated significant differences in the CLL and CLL + OM groups. In addition, the frequency of OM diagnosis after CLL treatment was higher in the CLL + OM group compared to the CLL group.

In this study, the age at diagnosis of CLL was found to be lower than previously reported.^[1]^ It was thought that this situation might be related to racial or ethnic differences. In previous studies, it has been shown that the incidence of CLL and the age of diagnosis may vary from race to race. For instance, CLL incidence is about 5- to 10-fold lower in Asians compared to other populations.^[15]^ Also, Asians with CLL are younger and often have atypical morphologic and immunologic features when compared to Caucasians with CLL.^[15]^ Since both Mediterranean and Middle Eastern characteristics are present in Turkish individuals, there is a need for more demographic-based multicenter investigations or database studies to clarify the characteristics of CLL patients.

We detected OM in approximately 2 out of every 5 CLL cases. When we grouped patients with respect to the presence/absence of OM, there was no significant difference between the 2 groups in terms of age and sex. In a study evaluating long-term CLL and OM, the presence of OMs was found in 36% of subjects. In the same study, male sex, advanced age, and low platelet count were determined to be associated with OM development.^[16]^ The smaller size of our patient group, younger age, and shorter follow-up may explain these differences. In addition, in a comprehensive retrospective study, it was determined that the risk of OM was increased in CLL patients, especially with respect to the development of head and neck cancers, melanoma, lung cancer, and Kaposi sarcoma.^[17]^ The most common cancers that had developed after CLL were identified as lung, prostate, colon, and breast cancer, respectively.^[17,18]^ Although the sample size was small in our study, the most common OMs demonstrated a relative similarity, as shown by the high frequency of breast, colorectal, prostate, lung, and skin cancers.

When we compared the hemogram parameters during the diagnosis of CLL, we found that WBC, lymphocyte, and hemoglobin values in the CLL + OM group were significantly lower compared to the CLL group. In a study evaluating the incidence of secondary malignancy in CLL patients receiving ibrutinib treatment, it was stated that CD8 + lymphocyte count reduced the risk of secondary cancer, and that quantitative lymphocyte count could be used as a potential biomarker for the risk of OM.^[6]^ Our data are in agreement with this study. Previous studies have largely focused on the demographic data of CLL cases with OM, rather than their laboratory and genetic parameters. Therefore, we could not find any studies to compare our laboratory data–other than lymphocytosis. This situation alone demonstrates the need for further studies on the subject.

The fact that the development of OMs was significantly more frequent in patients who received treatment after CLL diagnosis (in the CLL + OM group) was thought to be compatible with the increased risk of malignancy due to chemotherapy-induced DNA damage and immunosuppression, which is a relationship that has been repeatedly shown in prior studies.^[19–21]^ Also, similar to previous reports, hematologic malignancies associated with CLL were mostly observed to be myelodysplastic syndrome–AML–only after CLL treatment.^[8]^ Our group size was not large enough to analyze the effect of individual treatment regimens on the risk of OM, future studies may find value in analyzing subjects according to treatment. There are various studies assessing the risk of increased secondary malignancy in relation to the use of fludarabine, chlorambucil, cladribine, and Bruton kinase inhibitors for CLL therapy.^[5–8]^ However, new agents such as obinutuzumab, venetoclax, and idelalisib have been added to the arsenal of medications used for CLL treatment, and approaches are gradually but constantly changing. To the best of our knowledge, data are insufficient concerning the contribution of novel targeted therapy to the development and progression of OM. In the era of targeted therapy, the risk of secondary malignancies, which is the most important long-term side effect in determining mortality (as well as its effectiveness in the treatment of CLL), needs to be examined with multicenter studies.

In this study, the stages of CLL recorded at diagnosis were significantly different between the CLL and CLL + OM groups. Although this situation is thought to be associated with the immediate and intensive use of chemoimmunotherapy in advanced-stage patients,^[22]^ this should be confirmed by more detailed studies in which a larger number of patients are evaluated.

Another important detail that should be noted was the shorter survival time and the difference in death causes when the 2 groups were compared. In the CLL + OM group, patients died more frequently due to malignancy progression and sepsis/febrile neutropenia. It was thought that this might be due to the fact that the CLL + OM group consisted of cases with more advanced cancer who had received relatively intense chemotherapy/radiotherapy. Patients in this group may have been more susceptible to infection due to the greater immunosuppressive effects of intense chemotherapy regimens. This situation was also noted in various previous studies.^[23]^ Studies have established that chemotherapy use is associated with a greater likelihood of developing new malignancy in patients with CLL,^[7]^ but, to our knowledge, there are no studies focusing on the progression risk of OMs in patients with CLL. In research examining the effects of comorbidities on outcomes in CLL cases, it has been shown that the presence of comorbidity is associated with worse clinical outcomes.^[14,24]^ In addition, it was suggested in a study that there may be an interaction between the comorbidities seen in CLL cases and the treatment of the disease and that both conditions are associated with survival in CLL cases.^[25]^ The difference in survival time between the groups in our study is consistent with previous studies. It can be said that the presence of OM has a decreasing effect on the survival time of CLL cases.

The most important limitation of our study was its retrospective design and the inclusion of cases followed in a single center. Due to the retrospective design of the study, risk factors that may be associated with the development of OM could not be assessed. The number of patients was relatively low due to time and region limitations. For this reason, subgroup analyses could not be performed according to the type of CLL chemotherapy utilized. Although diagnosis times were recorded, the temporal relationship between CLL and OM could not be commented on, since features such as the onset of disease and early/late diagnosis were unknown.

CLL patients with OM had a more advanced CLL stage and the survival of these patients was significantly shorter. CLL cases without OM had higher WBC, lymphocyte, and hemoglobin levels. In addition, CLL-associated OM appears to occur more frequently in the post-treatment period. In cases with a diagnosis of CLL, care should be taken to detect OMs and it should be kept in mind that the presence of OMs may reduce survival. With future prospective studies, the causal relationship between OM and CLL can be revealed and the parameters affecting survival can be examined in more detail. These discoveries could then have the potential to influence treatment planning and may lead to the identification of management methods that can reduce OM likelihood in patients who are diagnosed with CLL.

## Author contributions

**Conceptualization:** Esra Turan Erkek.

Data curation: Eda Aslan.

Formal analysis: Eda Aslan.

Funding acquisition: Esra Turan Erkek.

Investigation: Esra Turan Erkek.

Methodology: Esra Turan Erkek.

Project administration: Esra Turan Erkek.

Resources: Esra Turan Erkek.

Software: Eda Aslan.

Supervision: Esra Turan Erkek.

Validation: Esra Turan Erkek.

Visualization: Esra Turan Erkek.

Writing – original draft: Esra Turan Erkek.

Writing – review & editing: Esra Turan Erkek.
